# Electrochemical Co-deposition of Polydopamine/Hyaluronic Acid for Anti-biofouling Bioelectrodes

**DOI:** 10.3389/fchem.2019.00262

**Published:** 2019-04-30

**Authors:** Semin Kim, Sanghun Lee, Junggeon Park, Jae Young Lee

**Affiliations:** ^1^School of Materials Science and Engineering, Gwangju Institute of Science and Technology, Gwangju, South Korea; ^2^Department of Biomedical Science and Engineering, Gwangju Institute of Science and Technology, Gwangju, South Korea

**Keywords:** polydopamine, hyaluronic acid, electrochemical, bioelectrodes, anti-fouling

## Abstract

Bioelectrodes are key components of electronic devices that efficiently mediate electrical signals in biological systems. However, conventional bioelectrodes often undergo biofouling associated with non-specific proteins and cell adhesion on the electrode surfaces, which leads to seriously degraded electrical and/or electrochemical properties. Hence, a facile and effective method to modify the surface of bioelectrodes is required to introduce anti-biofouling properties and improve performance. Here, we report an electrochemical surface modification of a bioelectrode via co-deposition of hyaluronic acid (HA) and polydopamine (PDA). The electrochemical polymerization and deposition of PDA offered simple and effective incorporation of highly hydrophilic and anti-fouling HA to the electrode surfaces, with no substantial increase in impedance. HA-incorporated PDA (PDA/HA)-modified electrodes displayed significant resistance to non-specific protein adsorption and the adhesion of fibroblasts. In addition, 4-week subcutaneous implantation studies revealed that the modified electrodes attenuated scar tissue formation compared with that induced by unmodified bare electrodes. This simple and effective electrochemical surface modification could be further employed for various implantable bioelectrodes (e.g., prosthetics and biosensors) and could extend their bioelectronic applications.

## Introduction

Recently, biomedical electronic devices have been developed for various applications, such as cardiac pacemakers, implantable prosthetic devices, and neural electrodes (Xu et al., 2014; Choi et al., 2016; Feiner et al., 2016; Park et al., 2018). In these devices, a bioelectrode plays an important role because it directly receives and/or transmits biological signals in contact with cells and tissues; therefore, its performance and biocompatibility are generally considered essential features for bioelectrode design and development (Fattahi et al., 2014; Kleber et al., 2017). However, for conventional implanted bioelectrodes, scar tissue is frequently formed around the electrodes, which eventually impedes their electrical and/or electrochemical properties, and thus limits their practical uses (Lin et al., 2013; Hu et al., 2016). This phenomenon is known as a typical foreign body response (FBR), which occurs as a host defense mechanism when foreign biomaterials are implanted into the body. The initial FBR involves non-specific protein adsorption onto the material surfaces, followed by attachment of various cells (e.g., macrophages and fibroblasts), and eventual scar tissue formation around the biomaterial implants (Grainger, 2013; KyungáKim, 2016). Therefore, a simple and effective strategy to develop anti-biofouling bioelectrodes is required to suppress scarring associated with the bioelectrodes and thus improve their functions in a biological milieu.

Several studies have reported the surface modification of electrodes using hydrophilic polymers to reduce the FBR. Hydrophilic polymers [e.g., hyaluronic acid (HA) and poly(ethylene glycol) (PEG)] on material surfaces hold a large amount of water and form hydration layers that can resist protein adsorption and/or denaturation and cell adhesion (Banerjee et al., 2011; Liu et al., 2016). To tether these polymers on the electrodes, chemical covalent modification is typically employed. However, most conductive electrode materials (e.g., metals and metal oxides) do not possess chemical functional groups to form covalent bonds with polymers (except for certain specific reactions, e.g., the thiol-gold reaction). For example, Brash's group coated gold electrodes with thiolated poly(ethylene glycol) by chemisorption and demonstrated the anti-fouling nature of the resultant surfaces (Unsworth et al., 2005). In addition, chemical reactions between electrodes and polymers for coating are difficult to be controlled precisely in term of the reaction degree and location. Plasma treatment has been utilized to generate functional groups on the surfaces for further coupling with various polymers; however, it possesses some disadvantages, such as the requirement of a complicated process, difficulties in controlling the coating degree and selective modification, and importantly, damage to whole devices (Cao et al., 2007; Chittrakarn et al., 2016; Park et al., 2019). By contrast, an electrochemical method for electrode passivation could be a good option because of the relatively easy control over the modification reaction, the lack of a requirement for toxic chemicals, and possibility of selective electrode coatings (Lee and Schmidt, 2010; Kim et al., 2017).

Hyaluronic acid (HA) is an anionic polysaccharide present in the body that has excellent hydrophilicity and biocompatibility (Highley et al., 2016). In particular, HA-based materials have been recognized as attractive anti-biofouling materials because of their excellent moisture-retention and lubrication ability, which properties are attributed to the amide (CO-NH) or carboxyl (-COOH) groups present in HA (Huang et al., 2015; Ye et al., 2016). However, HA cannot be directly employed for electrochemical deposition because it does not inherently contain electrochemically active groups. Moreover, HA binding with metallic substrates is generally poor. Consequently, a simple and effective method for the electrochemical immobilization of HA onto conductive electrode materials is required. Dopamine (DA) is a mussel-derived bio-adhesive molecule. In particular, its polymeric form [polydopamine (PDA)] exhibits strong adhesion to various substrates and thus has been explored extensively to tether various molecules (e.g., proteins and peptides) on various substrates (Lee et al., 2007; Ryu et al., 2018). PDA is usually obtained via a chemical oxidation reaction through self-polymerization in alkaline solution. The catechol group in DA moieties is oxidized to quinone, which can interact with various molecules via hydrogen bonding, hydrophobic interactions, and chemical reactions (e.g., Michael addition and Schiff base reactions) (Rai and Perry, 2012; Rodriguez-Emmenegger et al., 2013; Salazar et al., 2016). Importantly, a DA moiety is an electroactive molecule that can be oxidized and/or polymerized to PDA by applying oxidative potentials (Stöckle et al., 2014). Previously, we reported the electrochemical surface modification of electrodes using DA-HA conjugates (DA-HA) (Kim et al., 2017). Electrodes electrochemically coated with DA-HA exhibited non-fouling properties (resistance to non-specific serum protein adsorption and cell attachment) with no impairment of the electrodes' original electrochemical properties; however, that procedure required additional steps for DA-HA conjugation and purification.

**Figure 1:**
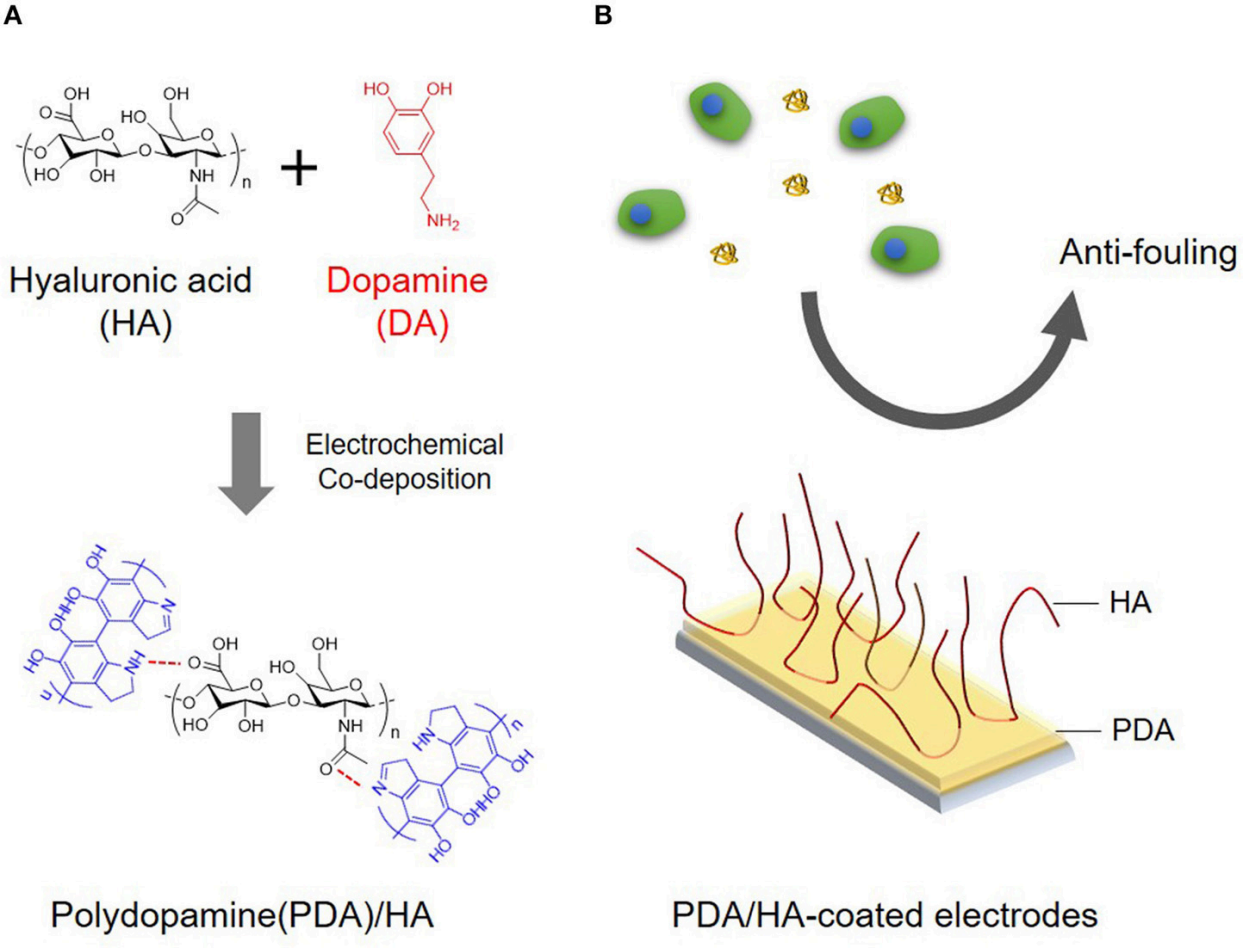
**(A)** Schematic illustration for electrochemical co-deposition of PDA/HA. **(B)** Scheme showing the use of anti-fouling of PDA/HA-coated electrodes.

In the present study, we aimed to develop a simple and effective method to create anti-fouling electrode surfaces via electrochemical coating of HA and PDA. Specifically, PDA was electrochemically deposited on the electrode surface in the presence of HA. We expected that HA would be incorporated into the PDA layer via entrapment during PDA deposition and/or its strong molecular interactions with PDA, displaying stable functions for biofouling resistance (Figure 1). The deposition of PDA and HA was studied using a quartz crystal microbalance (QCM). The PDA and PDA/HA-coated electrode surfaces were characterized by HA immunostaining, water contact angle (WCA) measurements, atomic force microscopy (AFM), and electrochemical impedance spectroscopy (EIS). Subsequently, we evaluated the electrode anti-fouling properties via a serum protein adsorption test, *in vitro* cell adhesion, and *in vivo* compatibility test.

## Materials and Methods

### Materials

Dopamine hydrochloride (DA), potassium ferrocyanide (K_4_Fe(CN)_6_), and fluorescein-isothiocyanate-labeled bovine serum albumin (BSA-FITC) were purchased from Sigma Aldrich (St. Louis, MO, USA). Hyaluronic acid (1.5 × 10^6^ Da) was obtained from LG Chem (Daejeon, Republic of Korea). Dulbecco's modified Eagle's medium, Dulbecco's phosphate buffered saline, and fetal bovine serum (FBS) were purchased from Gibco (Grand Island, NY, USA). An FITC-labeled HA binding peptide (FITC-HABP) was purchased from ChinaPeptides (Shanghai, China).

### Electrochemical Coating of HA-Incorporated PDA on Electrodes

Indium tin oxide-coated slides (ITO electrodes; AMG, Gwangju, Republic of Korea) were washed by successive sonications in acetone and methanol for 10 min each. The polymerization solution [10 mM DA and 2 mg/mL of HA in phosphate-buffered saline (PBS, pH 6)] was freshly prepared. For electrochemical deposition, a potentiostat (VersaSTAT3 electrochemical working station, Princeton Applied Research, Oak Ridge, TN, USA) was used in a three-electrode system with a Pt wire as the counter electrode and a standard calomel electrode (SCE) as the reference electrode. PDA/HA deposition was performed on the ITO electrodes by applying a constant potential of 1 V for 30 or 60 min. The mass change profile and current profile were monitored during electrochemical deposition using a quartz crystal microbalance (QCM; QCM922A, SEIKO EG&G, Tokyo, Japan). After coating, the samples were washed with double de-ionized (DDI) water and then stored in PBS (pH 7.4). For comparison, PDA was also deposited on ITO electrodes in a HA-free polymerization solution [10 mM DA in PBS (pH 6)] under the same electrochemical conditions as the PDA/HA deposition.

### Florescence and Image Analysis

The presence of HA on the modified electrode surfaces was studied by immunostaining with the FITC-HABP. Samples were incubated in blocking solution [1% BSA in PBS (pH 7.4)] at room temperature for 30 min, followed by incubation in FITC-HABP [5 μg/mL in PBS (pH 7.4)] at room temperature for 45 min. The samples were washed with PBS, and fluorescence images were acquired using a ChemiDoc MP image system (Bio-Rad, Hercules, CA, USA) with the same exposure time for all samples.

### Atomic Force Microscopy

An atomic force microscope (AFM; XE-100, PARK System, Suwon, Republic of Korea) was used to characterize the surface morphology and thickness of the coated films. Substrates were scanned in a tapping mode using a non-contact, high resonance tip (Nanoworld, Neuchâtel, Switzerland) at a scan rate of 0.3 Hz. The thicknesses of the PDA and PDA/HA films was measured using a DEKTAK-XT stylus profiler (Bruker, Karlsruhe, Germany).

### Water Contact Angle Measurement

Static water contact angles of the samples were measured using a contact angle analyzer (Phoenix 300, Surface Electro Optics Co., Suwon, Republic of Korea). A 10-μL DDI water droplet was dropped onto the sample at room temperature.

### Electrochemical Impedance Spectroscopy

Electrochemical impedance spectroscopy (EIS) was performed on bare ITO and PDA/HA-modified ITO electrodes. For EIS analysis, ITO electrodes were covered with a tape having a circular hole (6 mm in diameter) before electrochemical deposition to ensure a constant electrode area (0.2829 cm^2^). The same three-electrode system as described in section Electrochemical Coating of HA-Incorporated PDA on Electrodes was employed, using a VersaSTAT3 electrochemical working station. An alternative sinusoidal potential of 10 mV and a DC potential of 0 V (vs. open circuit voltage) was applied in a range of 10^−1^-10^5^ Hz and the measurements were performed in PBS with 5 mM [Fe(CN)_6_]^3−/4−^.

### Protein Adsorption Test

A QCM was used to monitor the protein adsorption onto the bare, PDA- and PDA/HA modified ITO electrodes. The electrodes were exposed to the solutions pumped at 100 mL/min for 1 h in a QCM flow cell. In the order of PBS, 10% FBS, and PBS, individual solutions were flowed over the electrodes. The frequency changes were observed using the WinQCM software. The effects of protein adsorption onto the electrode samples in terms of electrochemical impedance were studied. Bare and modified electrodes were incubated in 10% FBS in PBS (pH 7.4) at 37°C for 1 h. The impedance spectra from the electrodes before and after protein adsorption were obtained in frequency ranges of 1–10^5^ Hz, with an amplitude of 5 mV. Nyquist plots of the impedance data were fitted with an equivalent circuit using the ZsimpWin 3.21 software package (Princeton Applied Research). The charge transfer resistance (R_ct_) was calculated from fitting the impedance data and then compared among the electrodes.

### *In vitro* Cell Culture

To study the ability of PDA/HA-modified substrates to resist cell attachment, mouse NIH3T3 fibroblasts were seeded and cultured on bare, PDA-, PDA/HA-coated ITO electrodes, respectively. Fibroblasts were maintained in a tissue culture plate with 5% CO_2_ at 37°C in a culture medium consisting of Dulbecco's modified Eagle's medium (Gibco), 10% heat-inactivated FBS, and 1% antibiotic/antimycotic solution. Before cell seeding, the samples were sterilized by exposure to ultraviolet light for 1 h. Fibroblasts were seeded at a density of 3 × 10^4^ cells per cm^2^ and incubated in a humidified incubator with 5% CO_2_ at 37°C for 3 days.

### *In vivo* Tissue Compatibility

All animal experiments were performed with permission from the committee on animal research and ethics in Gwangju Institute of Science and Technology, Republic of Korea (Approval number: GIST-2017-044). *In vivo* animal experiments were conducted with BALB/c mice (6 weeks old) to assess the tissue compatibility of PDA/HA-modified electrodes. The PDA/HA-coated electrodes were first immersed in 70% ethanol for sterilization for 1 h, and then thoroughly washed with sterile Dulbecco's PBS. The back of each mouse was shaved and the shaved area was disinfected. The mice were anesthetized using 2% isoflurane. It should be noted that gold-coated epoxy electrodes were used for the *in vivo* study. In brief, SU-8 100 (MicroChem, Westborough, MA, USA) was spin-coated on a clean glass substrate at 1,500 rpm, followed by pre-curing (30 min at 65°C and 30 min at 95°C), ultraviolet light exposure (1 min), baking (90 min at 95°C), and developing (60 min in isopropyl alcohol). Then, a 50 nm-thick gold layer was coated on the fabricated epoxy probe using a sputter technique. The gold-coated epoxy electrode was peeled-off from the glass substrate and electrochemically deposited with PDA or PDA/HA. Samples were implanted in subcutaneous pockets made to the right and left sides of a midline incision on the mouse's back, and the skin was sutured using silk thread. Four mice were used for each sample—bare, PDA, PDA/HA. At 4 weeks after implantation, the mice were sacrificed. Skin and muscle tissues surrounding the samples were fixed in 3.7% formaldehyde and embedded in paraffin. The tissue blocks were sectioned to 5-μm-thick slides using a microtome. For histological analyses, hematoxylin and eosin staining and Masson's trichrome staining were performed according to the manufacturer's protocol (Sigma Aldrich, St. Louis, MO, USA). Images of stained tissues were acquired under an optical microscope.

### Statistical Evaluation

All tests were performed at least in triplicate and the results are reported as the mean ± standard deviation (SD) unless otherwise noted. Results between groups were analyzed by one-way ANOVA analysis using Origin software (Microcal Software, Inc., Northampoton, MA, USA), with appropriate corrections, such as Tukey's *post hoc* tests, for comparisons.

## Results and Discussion

### Electrochemical Deposition of PDA/HA

**Figure 2:**
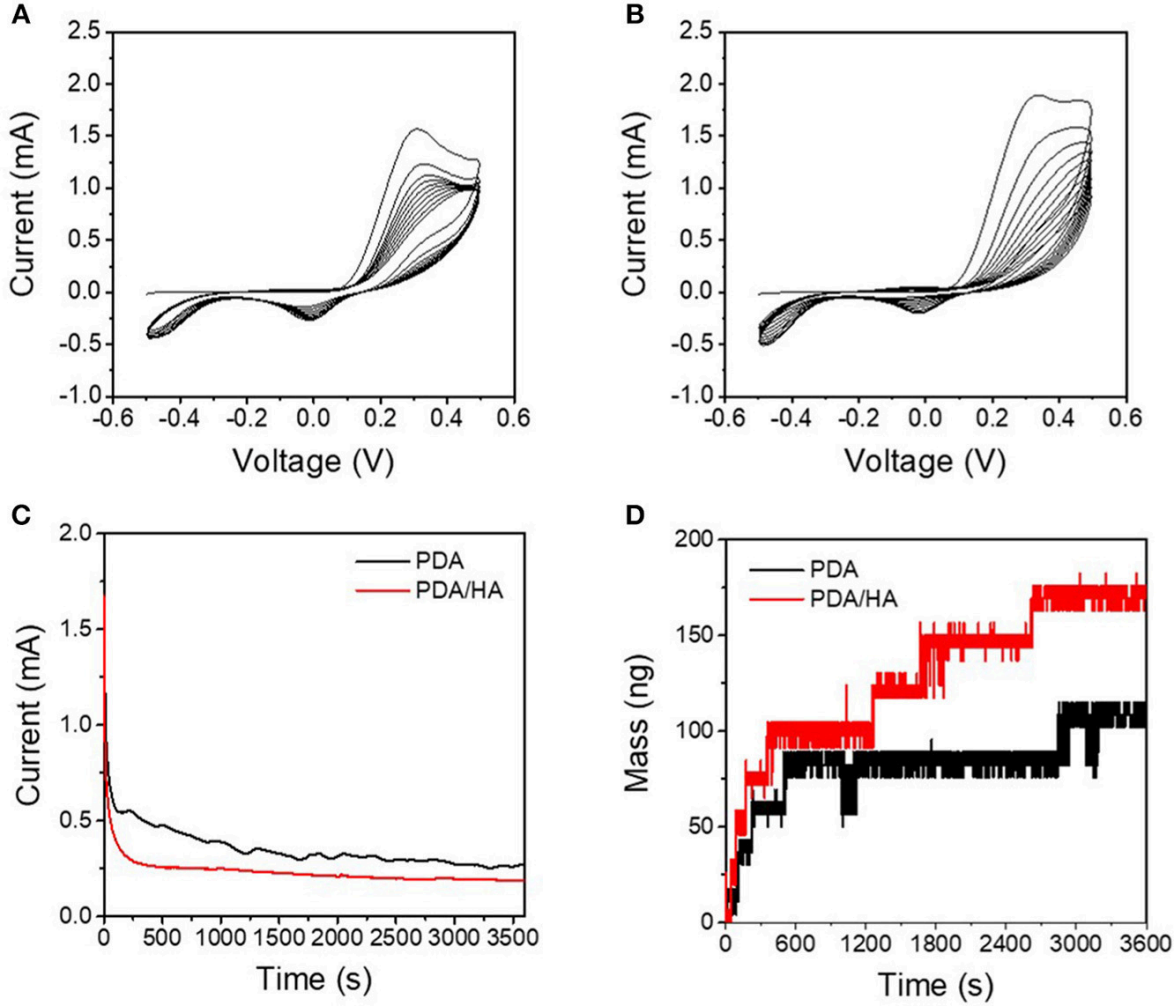
**(A)** Cyclic voltammograms of DA solution [10 mM DA in phosphate-buffered saline (PBS) (pH 6)] at a scan rate of 0.02 V s^−1^. **(B)** Cyclic voltammograms of DA/HA solution [10 mM DA and 2 mg/mL HA in PBS (pH 6)] at a scan rate of 0.02 V s^−1^. **(C)** Chronoamperograms of the DA solution and DA/HA solution at 1 V vs. standard calomel electrode (SCE). **(D)** Electrochemical quartz crystal microbalance (QCM) profiles during chronoamperometry (at 1 V *vs*. SCE) of the DA solution and the DA/HA solution.

The redox properties of DA and DA/HA were first examined to study the electrochemical deposition of PDA/HA by cyclic voltammetry in the voltage range from −0.5 V to 0.5 V vs. SCE (Figures 2A,B), before electrochemical deposition. DA and DA/HA exhibited distinct oxidation peaks at 0.2–0.4 V and reduction peaks at around 0 V, indicating the redox couples of DA/dopaquinone, and oxidation peaks at −0.1 V and reduction peaks around −0.5 V, indicating the redox couples of leucodopaminechrome/dopaminechrome. In particular, the DA/dopaquinone intermediate state is known to play an important role in adhesion to various material surfaces because of its ability of form bidentate and binuclear complexes on substrates. As the polymerization proceeded during multiple cyclic voltammetry scans, the currents for both PDA and PDA/HA polymerization steadily decreased, which indicated the part formation of insulating PDA domains on the electrode surfaces and deposition of insulating bulky HA molecules with PDA domains on the electrodes. We further investigated the electrochemical deposition of PDA and PDA/HA in the galvanostatic mode by applying a constant potential of 1 V (vs. SCE) for 1 h (Figure 2C). The current decreased over time during electrochemical deposition, likely caused by the gradual formation of the PDA and PDA/HA layer and the local depletion of DA and HA in the vicinity of electrode surfaces. Interestingly, PDA/HA showed a lower current than PDA, which indicated that the HA was entrapped in the PDA and acted as a barrier on the electrode surface. Furthermore, the QCM results indicated that the mass increased steadily for both PDA and PDA/HA during electrodeposition (Figure 2D). The mass of PDA/HA increased more than that of PDA, confirming that bulky HA was well entrapped in PDA. Thus, the results indicated successful electrochemical deposition of PDA/HA onto the electrodes.

### Characterization of PDA/HA-Coated Electrodes

**Figure 3:**
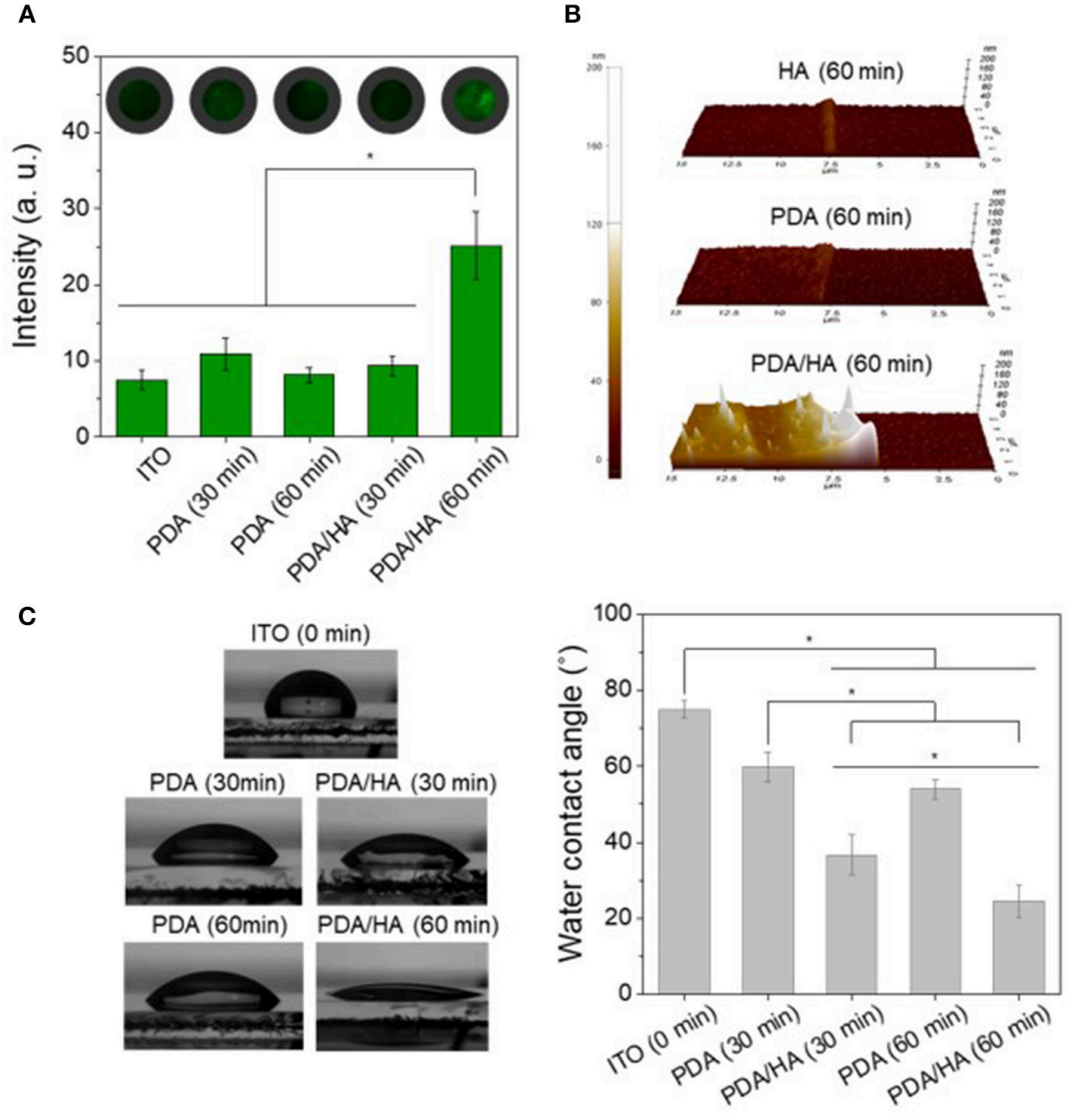
**(A)** Immunofluorescence images and fluorescence intensities of the various electrodes modified with PDA or PDA/HA. FITC-HABP (fluorescein isothiocyanate labeled hyaluronic acid binding peptide) was used for HA staining. Fluorescence images for each condition were randomly selected (*n* > 3) and intensities were calculated. **(B)** AFM images of HA (60 min), PDA (60 min), and PDA/HA (60 min)-coated electrodes. **(C)** Water contact angles of bare ITO, PDA (30 min, 60 min), and PDA/HA (30 min, 60 min)-coated ITO. Asterisks indicate statistical significance (*p* < 0.05).

The Electrodes electrochemically modified with PDA/HA were characterized by multiple techniques, such as immunostaining of HA, AFM, and WCA measurement. First, FITC-HABP conjugates were employed for immunostaining of HA to confirm the presence of HA on the various modified electrodes, including bare ITO, PDA, and PDA/HA with deposition times of 30 min or 60 min (Figure 3A). Bare ITO, PDA (30 min), PDA (60 min), and PDA/HA (30 min) had similar fluorescence intensity values. By contrast, PDA/HA (60 min) showed an approximately 3.4-fold higher fluorescence intensity than the bare ITO, indicating the successful introduction of HA chains between the PDA layers during electrochemical deposition. In addition, a high amount of free HA was deposited onto the PDA/HA surfaces, apparently via electrochemical co-deposition with PDA.

AFM was employed to assess of the film thicknesses and topographies of the various prepared coatings (Figure 3B). The average thicknesses of HA (60 min), PDA (60 min), and PDA/HA (60 min) were 5.9, 10.7, and 51 nm, respectively (Figure S1). The increased thickness of PDA/HA compared with PDA can be attributed to the incorporation of HA chains. Furthermore, the measured average roughness (Ra) of PDA and PDA/HA-coated ITO were 3.4 and 8 nm, respectively. The PDA/HA coated surface was slightly rougher than the PDA-coated surface, probably because of the entrapment of HA. Thus, it is possible to obtain a PDA/HA coated electrode, which is expected to be more efficient for proteins and cell resistance than bare and PDA-coated electrodes.

The hydrophilicity of the PDA/HA-coated electrode surface was evaluated by measuring the WCA (Figure 3C). The bare ITO surface was relatively hydrophobic, with a WCA of 74.9 ± 2.2° compared with the PDA and PDA/HA-coated electrodes. The PDA-coated ITO electrodes were more hydrophilic than ITO, displaying a WCA of 59.8 ± 3.9° for PDA (30 min) and 53.9 ± 2.7° for PDA (60 min). Compared with the bare and PDA-coated ITOs, electrochemical deposition of PDA/HA significantly decreased the WCA to 46.7 ± 9.7° and 24.5 ± 4.4° for PDA/HA (30 min) and PDA/HA (60 min)-coated electrodes, respectively. The increased electrochemical deposition time of PDA/HA resulted in slight increases in surface hydrophilicity of the PDA/HA-modified electrodes. The significantly hydrophilic surfaces of the PDA/HA-modified electrodes could be explained by successful incorporation of highly hydrophilic HA molecules into the coating. Highly hydrated and dynamic HA moieties immobilized on the coated layer were expected to play a key role in preventing the access and non-specific adsorption of proteins and cells by forming a hydration layer on the electrode surfaces. One-week incubation of the PDA/HA-modified electrodes in PBS revealed no significant changes in WCA, suggesting the HA component in the layer appeared to stably remain on the surfaces (Figure S2).

### Electrochemical Characterization of PDA/HA-Coated Electrodes

**Figure 4:**
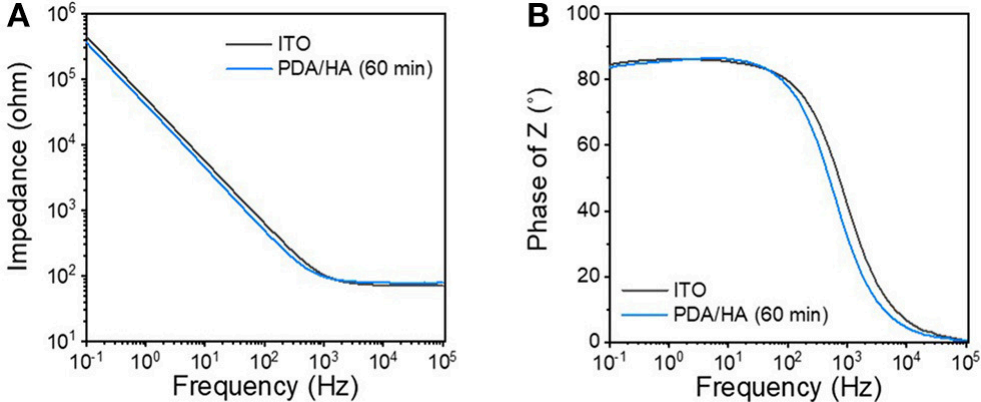
**(A)** Bode and **(B)** Nyquist plots of the bare ITO and the PDA/HA (60 min)-coated ITO electrodes.

Electrochemical impedances were analyzed as an important factor for the functions of the modified electrodes of biomedical applications (Figure 4). Impedance at low frequency (10^−1^-10^3^ Hz) was slightly decreased after PDA/HA coating; however, the difference between the unmodified bare ITO and the PDA/HA-modified ITO was not significant. This result might be attributed to the contribution of anionic HA at the interfaces and that the coverage by PDA/HA would not be too dense to prevent electron transfer reactions at the interfaces. Previously, we found that electrochemical deposition of DA-HA conjugates on ITO electrodes did not affect their impedances while displaying resistance to cell attachment (Kim et al., 2017). Moreover, we examined the stability of the modified electrodes while incubating them in PBS for 7 days. Impedances were not significantly altered during the incubation, implying that the PDA/HA modified electrodes would be stable under physiological conditions (Figure S3).

### Protein Adsorption Test

**Figure 5:**
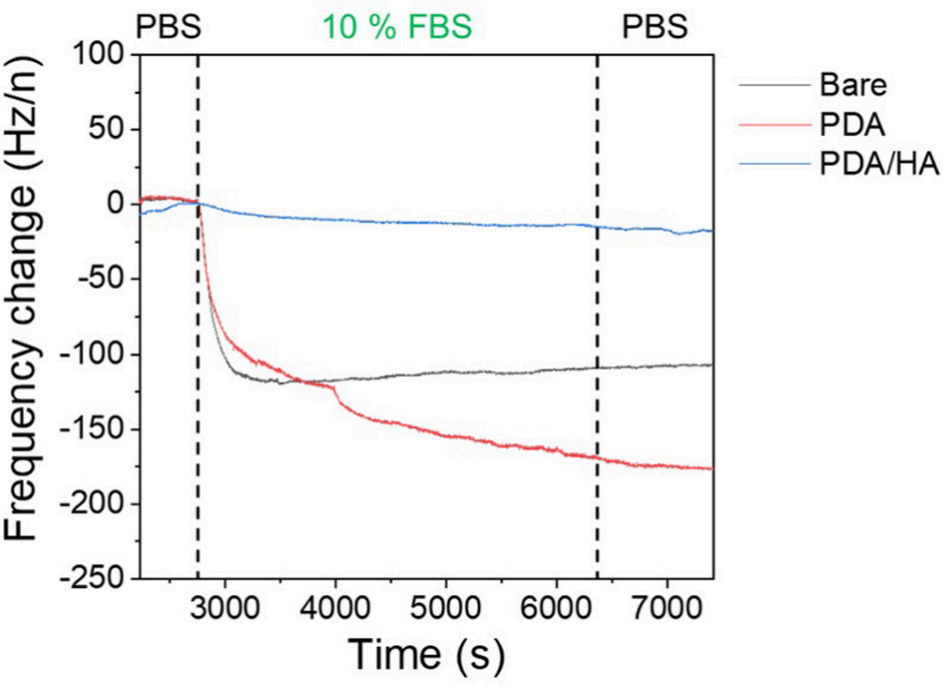
Protein adsorption on bare ITO, PDA (60 min), and PDA/HA (60 min)-coated ITO electrodes using a quartz crystal microbalance (QCM). Frequency change profiles of bare ITO, PDA (60 min), and PDA/HA (60 min)-coated electrodes during incubation in 10% fetal bovine serum (FBS) solution. Electrodes were flowed with phosphate-buffered saline (PBS), followed by 10% FBS solution for 3,000 s and rinsing with PBS.

**Figure 6:**
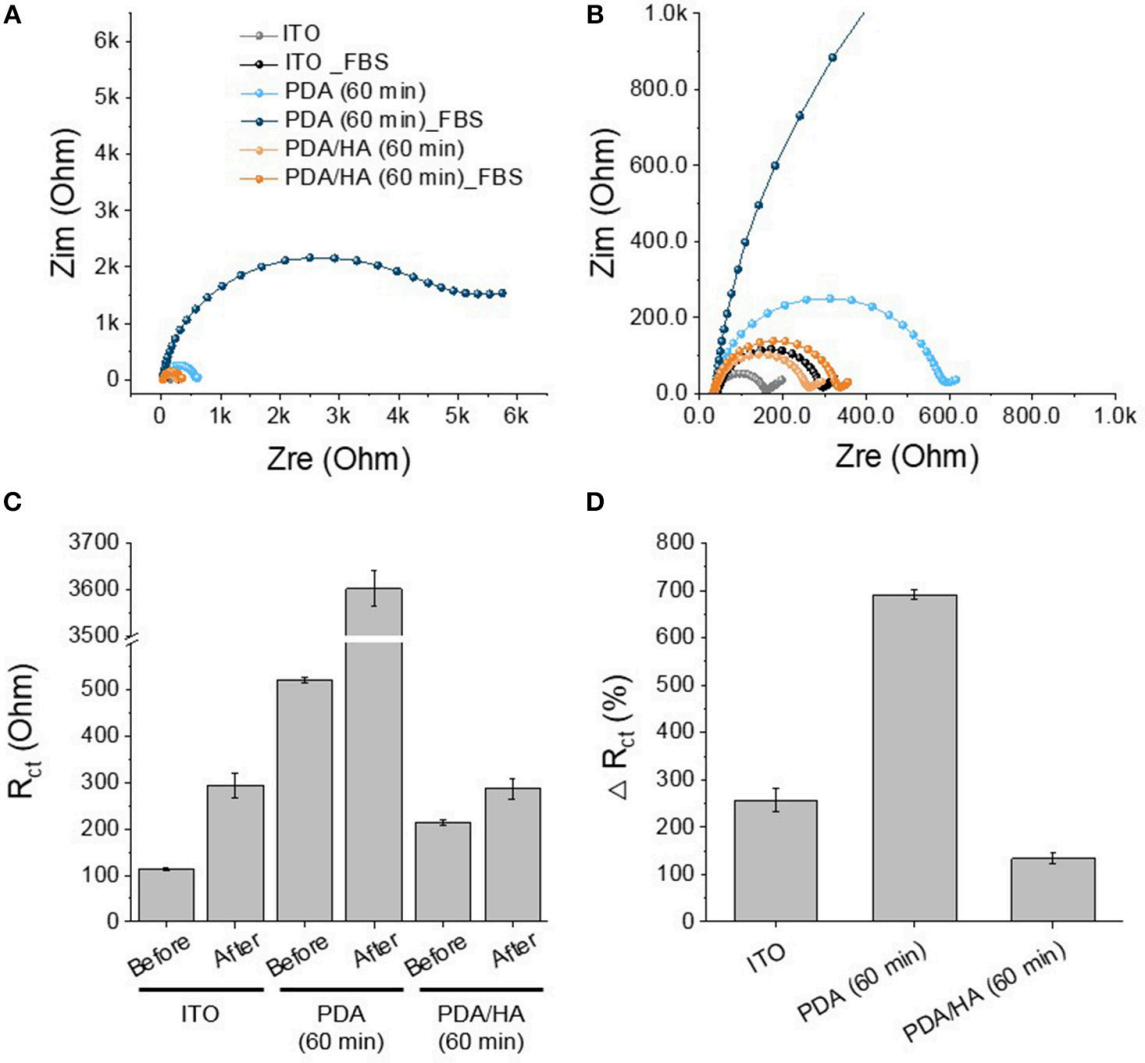
Protein adsorption test of PDA (60 min)-modified, PDA/HA (60 min)-modified, and bare ITO. **(A)** The Nyquist plot of the electrodes after incubation in 10% FBS solution for 1 h. **(B)** Magnified plot of **(A)**. **(C)** R_ct_ values of the electrodes before and after the incubation in 10% FBS solution. **(D)** Relative increases in R_ct_ values after incubation.

Protein adsorption prompts cell attachment and is the initial event that causes an eventual FBR of implanted biomaterials, including bioelectrodes. A QCM flow cell was used to explore non-specific protein adsorption onto the surfaces of the bare, PDA-, and PDA/HA-coated electrodes in the 10% FBS-containing PBS. Before exposure to FBS, PBS was flowed on the electrode surfaces until a stable frequency was observed. As shown in Figure 5, bare and PDA-coated electrodes showed significant mass increases when the FBS solution began to flow across the samples. Especially, the serum proteins were remarkably adsorbed onto the PDA-coated electrodes, likely because of strong molecular interactions between PDA and various substances, including proteins. In our previous report, we found a similar trend of higher protein adsorption to PDA/polypyrrole-modified gold electrodes than to polypyrrole alone (Kim et al., 2018a). By contrast, the PDA/HA-coated electrodes did not show significant mass changes. The reduced protein adsorption onto the PDA/HA-coated materials could be accounted for by the presence of HA chains that may hinder the access and interactions of proteins with the electrode surfaces. Furthermore, we performed electrochemical analyzes using redox probes, as a model for electrochemical sensing, to investigate the effects of non-specific protein adsorption on the electrical properties of the electrodes. Specifically, after the samples were incubated in 10% FBS solution for 1 h at 37°C, their electrical properties were analyzed in the presence of 5 mM [Fe(CN)_6_]^3−/4−^. Non-specific protein adsorption typically inhibits the current flow of the redox probe at the electrode/electrolyte interface, resulting in increased charge transfer resistance (R_ct_) of the electrodes and inconsistent signals, which is critically related to the practical applicability of bioelectrodes and biosensors. As shown Figure 6, most samples showed significant increases in R_ct_ values after incubation with serum proteins. The R_ct_ values of the bare ITO, PDA (60 min), and PDA/HA (60 min) electrodes increased from 114, 521, and 214, to 293, 3,602, and 286, respectively. Importantly, the PDA/HA-modified electrode showed a lower increase in R_ct_ than the bare and PDA-modified electrodes. The relative increases in R_ct_ values between before and after protein adsorption were 258 ± 23.9%, 692 ± 10.3%, and 134 ± 11% for bare ITO, PDA (60 min), and PDA/HA (60 min)-modified electrodes, respectively. For the PDA (60 min)-modified samples, which showed the highest protein adsorption, the R_ct_ increased by approximately 43 times post incubation compared with that of PDA/HA. These results are consistent with previous studies that PDA promotes protein adsorption (Hecker et al., 2018). The resistance changes of the PDA/HA (60 min)-modified electrode was relatively small compared with those of the other samples. These EIS results suggested that HA entrapped in the PDA layer could prevent fouling and improve the functions of implantable bioelectrodes. The result, as mentioned above, suggests that the PDA/HA coating does not cause any changes in electrochemical properties of the bioelectrodes, and in this respect, the PDA/HA-modification can be widely used in bioelectronics applications.

### *In vitro* Cell Culture Studies

**Figure 7:**
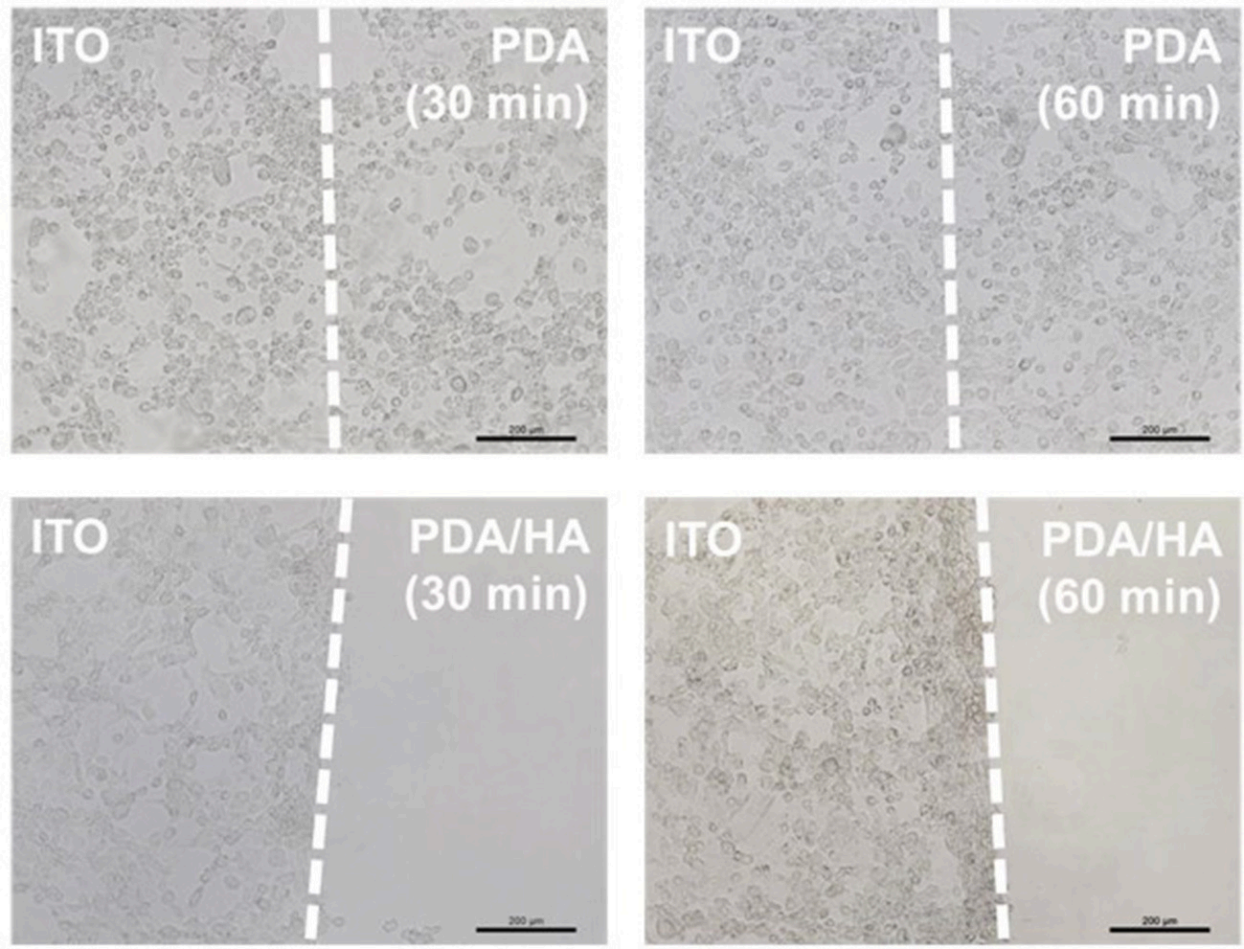
Optical micrographs of fibroblasts cultured on the various electrodes electrochemically modified with PDA or PDA/HA for different times. Dashed lines indicate the borders between unmodified ITO and modified areas on in individual electrodes. Scale bars are 200 μm.

Fibrous scar tissue encapsulation of the implanted electrodes can be caused by attachment of non-specific proteins and cells, such as macrophages and fibroblasts. To observe cell adhesion, fibroblasts were cultured *in vitro* on bare, PDA-, and PDA/HA-coated ITO electrodes. As shown in Figure 7, no fibroblasts were found on the PDA/HA-modified substrates, regardless of the deposition time (30 or 60 min); however, cells adhered well and grew on the bare ITO or PDA-modified substrates. The deposition time for PDA/HA did not influence their ability to resist cell adhesion. In addition, the cells on the patterned substrates did not migrate from an ITO area to the PDA/HA-coated area (border lines are marked with dashed lines). These results indicated that the PDA/HA modification effectively hinders cell attachment, likely because the PDA/HA coating does not allow access of small protein molecules and large objects, such as cells. By contrast, fibroblasts adhered to the PDA-coated electrodes in both samples, regardless of the deposition time, showing no differences compared with the bare ITO surface. Higher cell attachment onto PDA could be explained in a similar context as the protein adsorption test with QCM in section Protein Adsorption Test, which was also consistent with the findings of previous reports that protein adsorption and cell adhesion were markedly promoted on PDA (Ku et al., 2010; Ding et al., 2014; Chuah et al., 2015; Kim et al., 2018a). The hydrophilic HA component on the PDA/HA appeared to contribute non-fouling properties (e.g., resistance to non-specific protein adsorption and cell attachment). Likewise, direct hydrogel coating on bioelectrodes can improve the antifouling property. However, in many cases, the electrical characteristics of hydrogel-coated electrodes were greatly deteriorated because the hydrogel acted as an electrical insulator and a diffusion barrier (Jung et al., 2017; Tavakoli and Tang, 2017). This issue can be partly addressed by conductive polymer-based hydrogel for electrode modification. Although modification with conductive hydrogels can lead to improved electrical properties, their antifouling property was greatly diminished (Cheong et al., 2014; Naveen et al., 2017). In contrast, our PDA/HA-modified electrodes present both good electrical characteristics and an excellent antifouling ability; in this respect, the PDA/HA-modification can be widely used in bioelectronics applications.

### *In vivo* Tissue Compatibility

**Figure 8:**
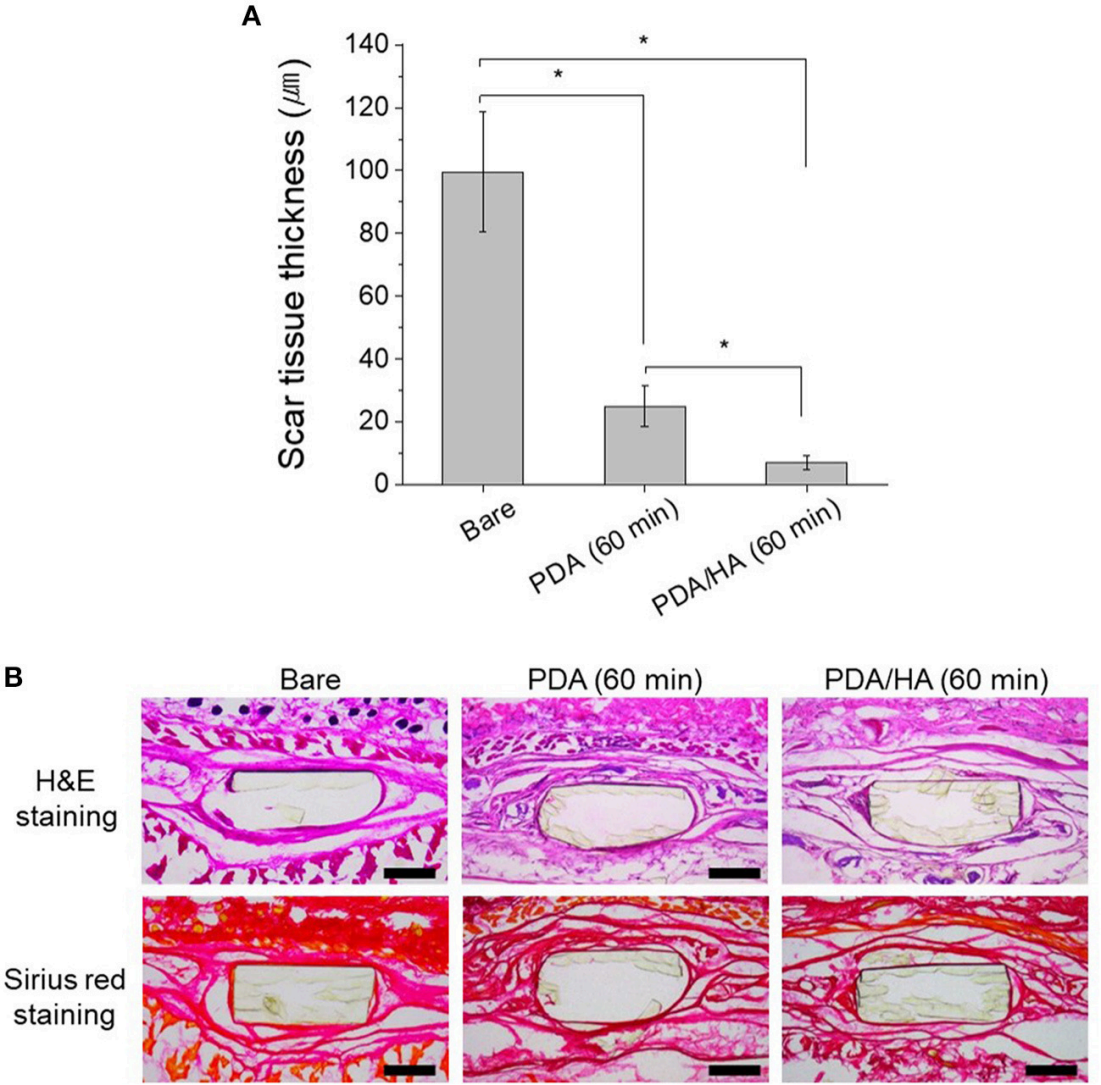
**(A)** Scar tissue thickness in the tissues implanted with bare, PDA (60 min), and PDA/HA (60 min)-modified electrodes. Asterisks indicate statistical significance (*p* < 0.05). **(B)** H&E and Sirius red staining images of the tissues after 4 weeks of subcutaneous implantation of bare, PDA (60 min), and PDA/HA (60 min)-modified electrodes. Scale bars represents 200 μm.

The tissue compatibility of PDA/HA was assessed using subcutaneous implantation of the samples for 4 weeks (Figure 8). No symptoms of severe tissue inflammation, such as redness or yellow fluids, were observed in all the tissues implanted with these samples. In the PDA/HA-coated electrode, the scar tissue thickness was significantly reduced compared with that measured for the bare and PDA-coated electrode. The scar tissue thicknesses were 99.5 ± 19.1, 25.1 ± 6.4, and 7.1 ± 2.3 μm for the bare, PDA-coated, and PDA/HA-coated samples, respectively. HA immobilization onto biomaterials was reported to mitigate inflammatory responses (Kim et al., 2018b). HA moieties could attenuate the non-specific adsorption of proteins and cells by passivating foreign materials, and the anti-inflammatory properties might further contribute the lack of activation of inflammatory responses to the PDA/HA-modified samples. Accordingly, PDA/HA-modified electrodes can be widely applied as biocompatible implant materials for various uses.

## Conclusions

To create anti-biofouling bioelectrodes, we developed and demonstrated a facile and effective method for direct electrochemical coating of PDA/HA onto electrodes. HA can be incorporated during the electrochemical deposition of PDA on the electrode surface. Characterization of the PDA/HA-modified substrates revealed the successful deposition of HA, creating a highly hydrophilic surface. Importantly, the electrochemical properties of the PDA/HA-modified electrode remained intact. Furthermore, the use of a PDA/HA coating significantly decreased the non-specific adsorption of serum proteins, cell attachment, and scar tissue formation *in vivo*. We believe that this simple and effective electrochemical method for electrode surface modification could benefit biocompatible biomaterials and thus could be used in diverse applications, including implantable bioelectrodes, biosensors, and prosthetics.

## Ethics Statement

All animal experiments were performed with permission from the committee on animal research and ethics in Gwangju Institute of Science and Technology, Republic of Korea (Approval number: GIST-2017-044).

## Author Contributions

SK, SL, and JL designed the study. SK and SL performed the study. JP assisted *in vivo* study. All authors participated in development and discussions.

### Conflict of Interest Statement

The authors declare that the research was conducted in the absence of any commercial or financial relationships that could be construed as a potential conflict of interest.
