# Supplementary material for: Electrochemical Co-deposition of Polydopamine/Hyaluronic Acid for Anti-biofouling Bioelectrodes
Source: Front Chem. 2019 Apr 30;7:262. doi: 10.3389/fchem.2019.00262 (PMC6503041; doi:10.3389/fchem.2019.00262)
Supplement: Supplementary file 1 [file Data_Sheet_1.docx]

Supplementary Material

Electrochemical co-deposition of polydopamine/hyaluronic acid for anti-biofouling bioelectrodes

**Semin Kim^1†^, Sanghun Lee^1†^, Junggeon Park^1^ and Jae Young Lee^1,2*^**

^1^ School of Materials Science and Engineering, Gwangju Institute of Science and Technology, Gwangju, Republic of Korea

^2^ Department of Biomedical Science and Engineering, Gwangju Institute of Science and Technology, Gwangju, Republic of

*** Correspondence:**Jae Young Lee
jaeyounglee@gist.ac.kr


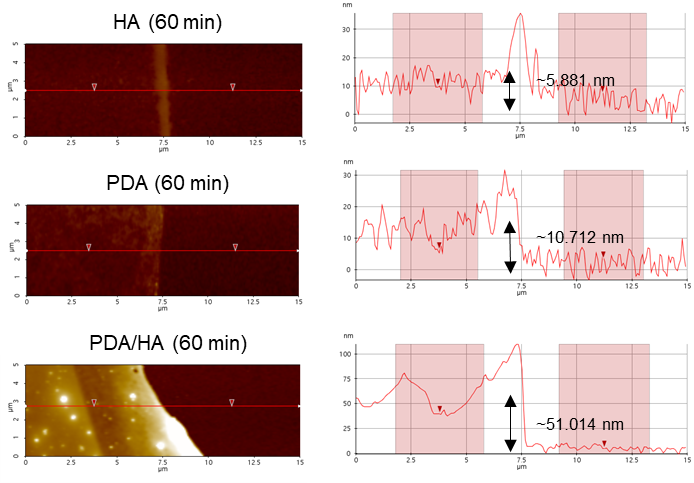


**Figure S1.** AFM images and line profiles of HA (60 min), PDA (60 min), and PDA/HA (60 min)-coated electrodes.


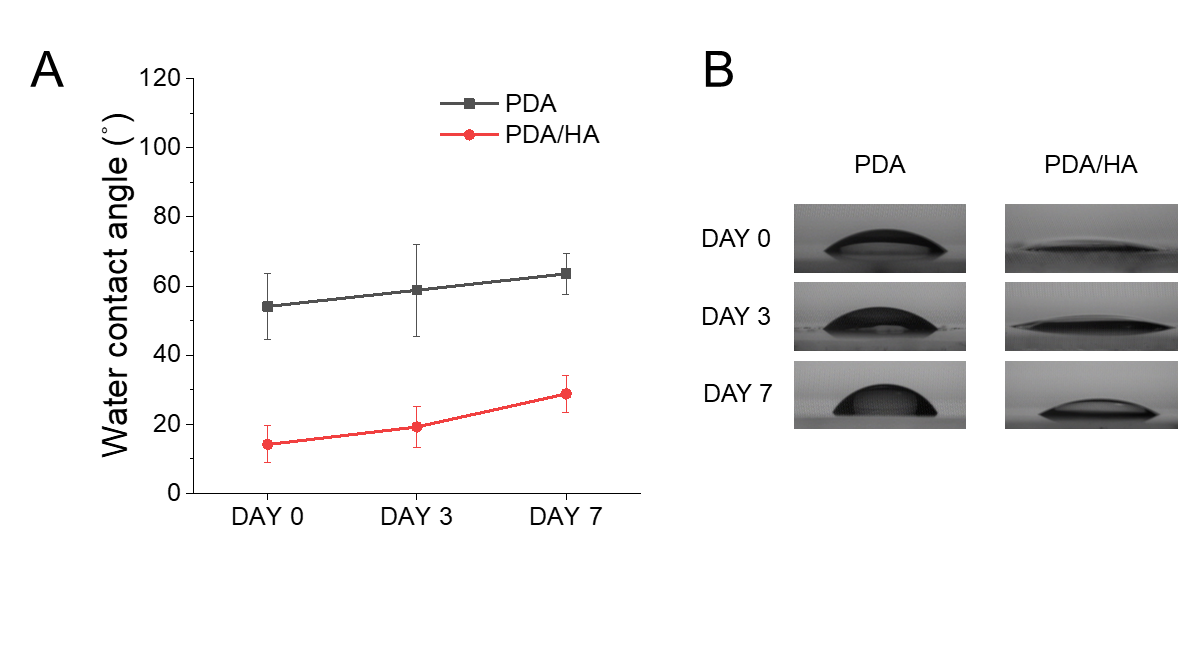


**Figure S2.** Water contact angle measurement of the PDA and PDA/HA-modified electrodes. The stability was evaluated by incubating the individual films at 37 °C on PBS (pH 7.4) for up to 7 days. (A) Water contact angles. No statistical difference among each group samples was found. (B) Photographs of the water droplets on different substrates.


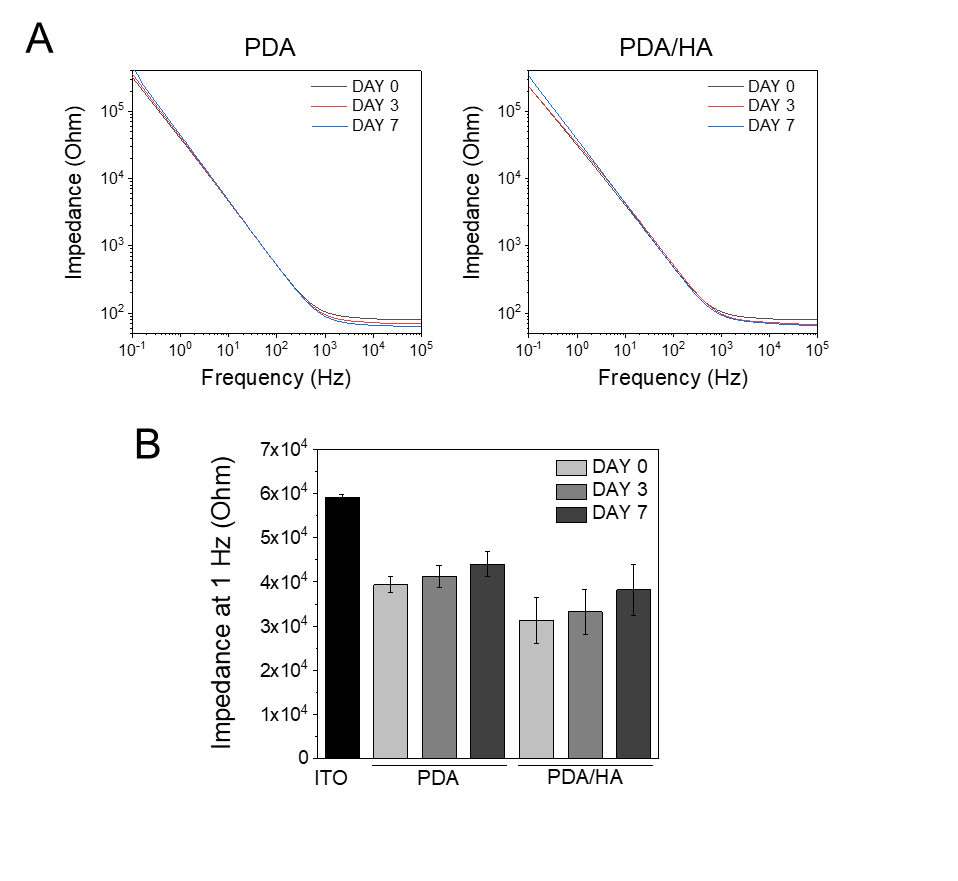


**Figure S3.** (A) Bode plots of the PDA, PDA/HA, and Hyaluronidase-treated PDA/HA-modified ITO electrodes for electrochemical stability. (B) Impedances at 1 Hz. Impedance spectra were collected in a range of 10^−1^ to 10^5^ Hz, applying an ac sinusoidal signal at 5 mV vs SCE, in PBS (pH 7.4). The stability was evaluated by incubating the individual films at 37 ° C in PBS (pH 7.4) for 7 days.
